# Isotopic systematics point to wild origin of mummified birds in Ancient Egypt

**DOI:** 10.1038/s41598-020-72326-7

**Published:** 2020-09-22

**Authors:** Marie Linglin, Romain Amiot, Pascale Richardin, Stéphanie Porcier, Ingrid Antheaume, Didier Berthet, Vincent Grossi, François Fourel, Jean-Pierre Flandrois, Antoine Louchart, Jeremy E. Martin, Christophe Lécuyer

**Affiliations:** 1 ENSL, CNRS, LGL-TPE, Univ Lyon, Univ Lyon 1, 69622 Villeurbanne, France; 2 Centre de Recherche et de Restauration Des Musées de France (C2RMF), Palais du Louvre, Porte des Lions, 14 quai François Mitterrand, 75001 Paris, France; 3 UMR 7055, Préhistoire et Technologie (Pretech), Université Paris Nanterre / CNRS, 21 allée de l’Université, 92023 Nanterre Cedex, France; 4 Laboratoire CNRS “Histoire et Sources des Mondes Antiques” (HiSoMA-UMR 5189), Maison de l’Orient et de la Méditerranée, Lyon, France; 5 Musée des Confluences, Lyon, France; 6 CNRS, ENTPE, UMR5023 LEHNA, Univ Lyon, Université Claude Bernard Lyon 1, 69622 Villeurbanne, France; 7 CNRS, UMR5558, Laboratoire de Biométrie et Biologie Évolutive, Univ Lyon, Université Lyon 1, 43 bd du 11 novembre 1918, 69622 Villeurbanne, France; 8 CNRS, Minist Culture, LAMPEA, Aix Marseille Univ, Aix-en-Provence, France

**Keywords:** Environmental social sciences, Biogeochemistry, Biogeochemistry

## Abstract

Millions of mummified birds serving for religious purpose have been discovered from archeological sites along the Nile Valley of Egypt, in majority ibises. Whether these birds were industrially raised or massively hunted is a matter of heavy debate as it would have a significant impact on the economy related to their supply and cult, and if hunted it would have represented an ecological burden on the birds populations. Here we have measured and analysed the stable oxygen, carbon and radiogenic strontium isotope compositions as well as calcium and barium content of bones along with the stable carbon, nitrogen and sulfur isotope composition of feathers from 20 mummified ibises and birds of prey recovered from various archeological sites of Ancient Egypt. If these migratory birds were locally bred, their stable oxygen, radiogenic strontium and stable sulfur isotopic compositions would be similar to that of coexisting Egyptians, and their stable carbon, nitrogen and oxygen isotope variance would be close, or lower than that of Egyptians. On one hand, isotopic values show that ibises ingested food from the Nile valley but with a higher isotopic scattering than observed for the diet of ancient Egyptians. On the other hand, birds of prey have exotic isotopic values compatible with their migratory behaviour. We therefore propose that most mummified ibises and all the birds of prey analysed here were wild animals hunted for religious practice.

## Introduction

A most significant feature of Egyptian religion was that most revered gods had the form of animals^1,2^. A common practice was the mummification of dead people, since the Old Kingdom (ca. 2,543–2,120 BC) until the fourth century AD, in order to preserve the body from decaying after death and reach the afterlife. From the New Kingdom (c.a. 1,539–1,077), and more particularly from the Late Period (ca. 722–332 BC) the animals have been also widely mummified, and that for four main purposes: victual mummies as food for the afterlife, sacred mummies to preserve the remains of a living incarnation of a god, pet mummies to provide an afterlife to beloved pets, and votive mummies as offerings to the gods. The widespread use of votive mummies to pray the gods Horus (depicted as a falcon) and Thoth (depicted as an ibis) led to the production of millions of bird mummies, as evidenced by archaeological discoveries from at least 38 catacombs across the Nile Valley^3^.

Such production of bird mummies, which can be considered as intensive, raised the question whether these birds were farmed, thus leading to a significant impact on the economy related to their supply, or hunted with an obvious impact on the birds populations dynamics. It has been speculated that ibises were extensively farmed^4^, a view supported by contemporaneous reports of such practices^5,6^. Moreover, finds of mummified ibises at all stages of development, from egg to juvenile to adult, support this suggestion. Ancient Egyptian textual references to « birth chapels » of ibises^7^ have also raised the question of artificial incubation of eggs for breeding purposes^8,9^, as well as to the fact that these birds are easy to rear according to ornithologists^3^. On the other hand, many wall scenes such as in the tomb of Nakht (New Kingdom) in the Theban Necropolis near Luxor show that mass bird hunting using clap-net traps was a common practice^10^. A mitogenomic study performed on extant and mummified ibises has shown a high level of genetic variation among mummified ibises comparable to that of extant wild ones, precluding the hypothesis of large-scale breeding^11^. We propose to use isotopic origin and diversity of food and water ingested by birds as an independent approach to investigate the wild or raised origin of mummified ibises and birds of prey. If the birds were raised along the Nile valley, they would drink waters from the Nile and eat food characterized by an isotopic variability similar to, or lower than that of the food consumed by contemporaneous Egyptians. Wild ibises and birds of prey such as the Greater Spotted eagle (*Clanga clanga*) or the Northern Long-legged buzzard (*Buteo rufinus rufinus*) are migratory birds. Therefore, it is expected that they would drink waters of various origins and consume preys of various nature. This hypothesis can be tested using stable oxygen, carbon, nitrogen, sulfur and strontium isotope compositions of bird organic (feathers) and mineral (bones) tissues that reflect the isotopic compositions of their foraging environment.

Indeed, bird bone apatite phosphate or carbonate has a stable oxygen isotope composition (δ^18^O) reflecting that of its drinking water with a fractionation that depends on species metabolism. By using the fractionation equation established between extant bird phosphate and its drinking water, it is possible to calculate the oxygen isotope composition of local surface waters ingested by birds^12^. In turn, the δ^18^O values of continental surface waters reflect their source (mainly precipitations^13–15^) and history (evaporation, refill^16,17^).

Carbon isotope composition (δ^13^C) of bird tissues reflect the δ^13^C value of its diet with a ^13^C-enrichment that depends on the digestive physiology and the type of analyzed tissue^18,19^. Feather-diet ^13^C-enrichment has been empirically determined between + 2.5 and + 3.8‰ for ibises^20^ and + 2.1‰ for falcons^18^, whereas apatite-diet ^13^C-enrichment between + 8.3 and + 11.6‰ was determined for carnivorous birds^21,22^.

Weathering of bedrock, marine aerosols and microbial processes have an influence on the δ^34^S values of environmental sulfur^23^. This isotopic composition is not affected by metabolism when incorporated into bird tissues, making it a geographic marker that has been intensively used for the study of extant birds’ migrations^24–26^**.**

Nitrogen in animal proteins is supplied almost entirely by dietary proteins in food. During their assimilation by animals, a fractionation between ^15^N and ^14^N occurs and lead to a feather-diet ^15^N-enrichment of 4.4 ± 0.6 ‰ for the White ibis *Eudocimus albus* and Scarlet ibis *Eudocimus ruber*^20^, and 2.7 ± 0.5 ‰ for the Peregrine falcon *Falco peregrinus*^18^. In that way, δ^15^N values of animal proteins can be used as an indicator of their trophic level^27^.

Environmental bio-available strontium in soils have radiogenic isotope ratios (expressed ^87^Sr/^86^Sr) that can also be used as a geographic origin tracer because of its large spatial isotopic variability that is largely controlled by geological substrates^28–30^. As in any plant or animal organisms, there is no diet-tissue isotopic fractionation of radiogenic strontium and ^87^Sr/^86^Sr ratios were successfully measured in bird tissues for provenance studies^31^.

Moreover, the duration of synthesis of organic or mineralized tissues and their remodeling rates reflect the period of life recorded in their stable isotope compositions. Bones have a very high turnover rate at year scale^32,33^, meaning that they record in their stable isotope compositions the last years of life of the bird. Feather growth rates vary depending on sex, environment (climate, food availability), species and type of feather^34^. Migratory birds have feather growth rates higher than sedentary species^35^. As a whole, feather growth rates varies from 1 to 7 mm per days^36^, so each piece of feather record two months of diet at most in their stable isotope compositions.

Based on these considerations, we interpreted the stable isotope compositions of mummified bird feathers and bones in terms of foraging environment prior to their death. If mummified birds were bred before mummification, they should have the same ^87^Sr/^86^Sr ratio than that of the sediments of the Nile Valley^37,38^, a drinking water δ^18^O_w_ value reflecting that of the Nile^39^, δ^34^S values within the range of vertebrates living in the Nile valley, as well as a variability in δ^15^N and δ^13^C values of their diet similar to, or lower than, that of the Egyptians living during the same period^40^.

## Materials and methods

### Sample collection

**Figure 1:**
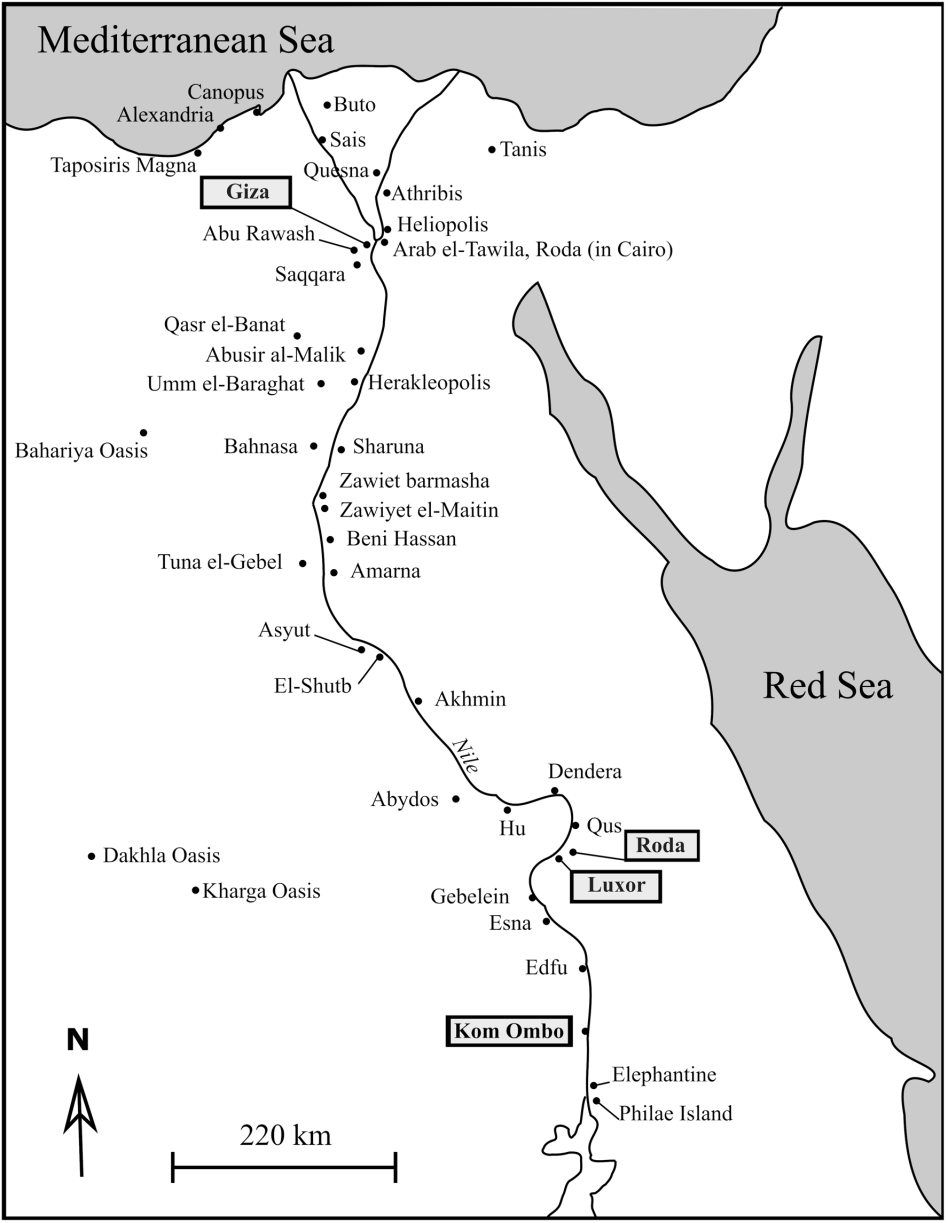
Localization of bird cemeteries in Egypt. Rectangles indicate the studied sites^3^. Map was drawn using INKSCAPE 1.0 (www.inkscape.org).

Feathers, bones and wrappings from 11 mummified ibises and 9 birds of prey have been sampled from the collections of the Musée des Confluences, Lyon, France. For conservation reasons, the selection of these birds was constrained by their preservation state and the accessibility of the bones and feathers under the wrappings, and only already unwrapped mummies could have been sampled. The precise origin of only four of these mummies was known (Giza, Kom Ombo, Luxor and Roda; Table S1 and Fig. 1).

Fragments were sampled from apparent long bone ends, ribs or scapulas. Feather fragments of a few centimeters were extracted from wings or tails, and incremental sampling of a single whole feather was realized for three specimens (Table S2).

### Analytical techniques

Bones were ground into fine powder with an agate mortar, and the organic matter was removed with 10% hydrogen peroxide for 15 min following the recommendations of Grimes and Pellegrini (2013)^41^. This treatment was selected for consistency purpose because it was used by Touzeau et al. (2013)^39^ to clean human and animal bone samples, the stable isotope compositions of which are used for comparison in this study. For the analysis of the oxygen isotope composition of bone phosphate (δ^18^O_p_), 30 mg aliquots of bone powder were treated using the procedure described in Lécuyer et al.^42^ consisting in isolating phosphate ions through bone dissolution and precipitation of silver phosphate. Five aliquots of 300 µg of silver phosphate from each sample were loaded into silver capsules along with the same amount of pure graphite. Isotopic ratios were determined using an IsoPrime isotope ratio mass spectrometer interfaced in continuous flow to a high temperature pyrolysis elemental analyser vario PYRO cube hosted by the platform “Ecologie Isotopique” of the ‘Laboratoire d’Ecologie des Hydrosystèmes Naturels et Anthropisés’ (LEHNA, UMR 5,023, Villeurbanne, France). Measurements have been calibrated against silver phosphate precipitated from the NBS120c (natural Miocene phosphorite from Florida), as well as with the NBS127 (Barium sulfate precipitated using seawater from Monterey Bay, California, USA). The value of NBS120c was fixed at 21.7‰ (V-SMOW) accordingly to Lécuyer et al.^43^, and that of NBS127 set at the certified value of 9.3‰ (V-SMOW) for correction of instrumental mass fractionation during CO isotopic analysis. Silver phosphate precipitated from standard NBS120c along with the silver phosphate samples derived from bird bioapatites was repeatedly analyzed (δ^18^O_p_ = 21.63 ± 0.14‰, n = 8) to ensure that no fractionation occurred during the wet chemistry procedure. Data are reported as δ^18^O values *vs*. V-SMOW (in ‰ δ units). Aliquots of the same calibrated material were measured at the beginning and the end of each batch to account for potential drift correction.

For the analysis of the oxygen and carbon isotope compositions of bone carbonate (δ^18^O_c_ and δ^13^C_c_), isotope ratios were determined using a PrecisION isotope ratio mass spectrometer interfaced in continuous flow to an iso FLOW system hosted by the platform “Ecologie Isotopique” of the ‘Laboratoire d’Ecologie des Hydrosystèmes Naturels et Anthropisés’ (LEHNA, UMR 5,023, Villeurbanne, France). Two aliquots of 2 mg of pretreated bone powder were loaded into glass vials and reacted with 10 drops of supersaturated orthophosphoric acid at 70 °C for one hour under a He atmosphere before starting 10 measurement cycles of the isotopic composition of the CO_2_ produced. The measured carbon and oxygen isotopic compositions were normalized relative to the Carrara marble (δ^18^O = − 1.84‰ V-PDB and δ^13^C = 2.03‰ V-PDB respectively;^44^ and the NBS18 (δ^18^O = − 23.20‰ V-PDB and δ^13^C = − 5.01‰ V-PDB ;^45–48^)). Reproducibility for the carbon and oxygen isotopic compositions of apatite carbonate is better than ± 0.05‰ and ± 0.1‰, respectively. Isotopic compositions of bone apatite carbonates are quoted in the standard δ notation relative to V-SMOW for oxygen and V-PDB for carbon.

Feathers of bird mummies are systematically covered by an organic balm^49^ used during the mummification process. Consequently, four cleaning procedures have been tested on five feathers of extant chicken and blackbird to check whether the cleaning reagents would have altered the primary isotopic compositions (see Supplementary data). The following cleaning protocol was applied to mummified feathers: each piece of feather was treated at first 2 times with a dichloromethane/methanol (3:1) solution and put in an ultrasonic bath for five minutes, then 2 times with a dichloromethane/acetone (3:1) solution and put in an ultrasonic bath for five minutes.

About 2 mg of clean feather fragments were loaded into tin capsules and their δ^13^C, δ^15^N and δ^34^S values were determined using an Isoprime100 isotope ratio mass spectrometer interfaced in continuous flow to a vario PYRO cube in combustion mode hosted by the platform “Ecologie Isotopique” of the ‘Laboratoire d’Ecologie des Hydrosystèmes Naturels et Anthropisés’ (LEHNA, UMR 5,023, Villeurbanne, France). Carbon, nitrogen and sulfur isotope compositions were calibrated against the following international standards: caffeine IAEA-600 (δ^13^C = − 27.77 ‰ V-PDB and δ^15^N = 1.0 ‰ AIR), sucrose IAEA-CH6 (δ^13^C = − 10.45 ‰ V-PDB), ammonium sulfate IAEA-N2 (δ^15^N = 20.3 ‰ AIR ajouter soufre), silver sulfide IAEA-S1 (δ^34^S = − 0.3 ‰ V-CDT) and barium sulfate NBS127 (δ^34^S = 20.3 ‰ V-CDT). Reproducibility for the carbon, nitrogen and sulfur isotopic compositions is better than ± 0.1‰, ± 0.3‰ and ± 0.2‰, respectively. Isotopic compositions of feather are quoted in the standard δ notation relative to V-PDB for carbon, to AIR for nitrogen and to V-CDT for sulfur. Besides official calibrated material, at the beginning of each NCS measurement campaign a set of in-house collagens is measured to ensure proper combustion of organic material as well as inorganic calibrated standards. The slopes of the respective calibration curves are used as a parameter to validate the results and no other quality control standards were used.

Bird bone apatite was dissolved and measured for elemental concentrations of strontium, barium and calcium using an inductively coupled plasma mass spectrometer (ICAP Q, Thermo Scientific). Measurement reliability was controlled through a set of blanks and standard NIST SRM-1486 (bone meal). Elemental concentrations are available in Table S1.

For the analysis of the strontium isotope composition of bone apatite, a 2 mg aliquot of bone sample was dissolved in concentrated nitric acid, purified through a strontium-specific resin (Sr-Spec Eichrom) and analyzed for ^87^Sr/^86^Sr ratios at the LGLTPE, ENS Lyon, with a Neptune Plus multicollector-ICP-MS (Thermo Scientific). A procedural blank underwent the same purification steps in the clean lab as the samples in order to control for possible Sr contamination and was then measured during the analytical session on the 23^rd^ of March 2019. The standard NIST SRM 987 was set to 14 V on mass 88 and the average voltage for all samples was adjusted at 10 V by diluting the purified Sr solutions. The undiluted procedural blank was measured at 0.0175 V on mass 88 and Sr contamination during purification is therefore considered negligible. The standard NIST SRM 987 was repeatedly measured between two samples and gave an average value of 0.710285 ± 0.000021 (2SD, n = 9). This value is close or nearly identical to previously published values (see review in De Muynck et al., 2009)^50^. Two measurements of NIST SRM 1486 bone meal gave a mean value of 0.709370, also close to the values reported in the literature^50^.

For radiocarbon of wrapping textiles^51–53^ and feathers, samples were first washed with ultrapure water, then with a mixture of methanol/dichloromethane (v/v 1/1), and finally with acetone in an ultrasonic bath for 10–15 min, in order to eliminate all organic contaminations (e.g. mummifaction balms). After the last treatment, samples were thoroughly rinsed three times with ultrapure water, in order to avoid solvent pollution of samples^54^. Finally, the cleaned samples were dried overnight under vacuum (100 mbars) at 65 °C. Samples were treated with the classic AAA method. This consisted of a series of washes at 80 °C for 1 h with a 0.5 N hydrochloric acid solution (HCl, VWR International), then with a 0.01 N sodium hydroxide aqueous solution (NaOH, VWR International), and once again with the 0.5 N HCl solution. Before each treatment, the supernatant was removed and the remaining fragments rinsed with water until neutrality of the washing waters was achieved. The treated samples were again dried overnight under vacuum (100 mbar) at 65 °C.

The dried organic fraction was then combusted 5 h at 850 °C under high vacuum (10^–6^ Torr). For this, 2 to 2.5 mg of pretreated sample were combusted in a quartz tube with 500 mg CuO [Cu(II) oxide on Cu(I) oxide heart for analysis, VWR International] and a 1 cm piece of Ag wire (99.95%, Aldrich). The combustion gas was separated by cryogenic separation and the CO_2_ was collected in a sealed tube. The graphitization was achieved by direct catalytic reduction of the CO_2_ with hydrogen, using Fe powder at 600 °C and an excess of H_2_. During the process, the carbon was deposited on the iron and the powder was pressed into a flat pellet. All measurements were performed at the Artemis AMS facility of Saclay, France^55^. ^14^C ages were calculated by correcting from the δ^13^C value, calculated from accelerator mass spectrometry (AMS) measurements of the ^13^C/^12^C ratio. Calendar ages were determined using OxCal v 4.2^56^ and the most recent calibration curve data for the Northern Hemisphere, IntCal13^51^. Calibrated age ranges correspond to 95.4% probability (2σ).

## Results

**Figure 2:**
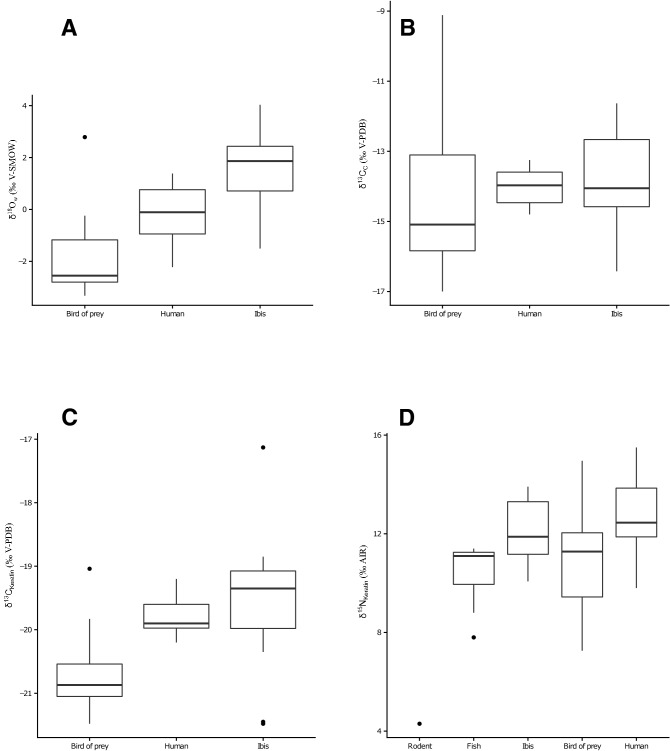
(**A**) Box-plot of the calculated water δ^18^O_w_ values ingested by the studied ibises and birds of prey compared to the published δ^18^O_w_ of the Nile water drunk by contemporaneous ancient Egyptian (n = 16)^39^. (**B**) Box-plot of the bone apatite carbonate δ^13^C_c_ values of studied ibises and birds of prey compared to that of contemporaneous ancient Egyptian bones (n = 15)^39^. (**C**) Box-plot of the δ^13^C_Keratin_ values of bird feathers compared to that of hair from contemporaneous ancient Egyptians (n = 5)^40^. (**D**) Box-plot of the δ^15^N_Keratin_ of bird feathers compared to that of hair from ancient contemporaneous Egyptians (n = 5), one rodent and 11 Nile perch scales^40^ (Table S4).

**Figure 3:**
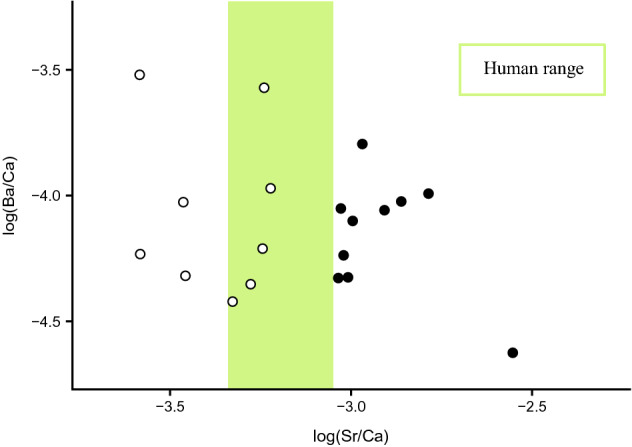
log(Ba/Ca) reported as a function of the log(Sr/Ca). Open circles = birds of prey and filled circles = ibises. The green band determines the range of log(Sr/Ca) values of ancient Egyptian^39^.

Stable isotope compositions of oxygen, carbon, nitrogen and sulfur, radiogenic isotope composition of strontium, and radiocarbon dating of bird apatite or feather keratin are given in tables S1, S2 and S3. From the many published isotopic studies of humans and animals from Ancient Egypt^57–61^, we added to our dataset stable isotope compositions of apatite oxygen and carbon as well as the carbon, nitrogen and sulfur isotope compositions of hairs, feathers or scales from contemporaneous and geographically close (within the Nile Valley) human and animals (Table S4 and S5). Bone oxygen isotope compositions of apatite phosphate range from 20.3‰ to 25.3‰ for ibises, and from 18.7‰ to 24.1‰ for the birds of prey (Table S1). Using the Bird phosphate-water fractionation equation^12^, calculated δ^18^O_w_ values of waters ingested by the birds range from − 1.5‰ V-SMOW to + 4.0‰ V-SMOW for ibises, and from − 2.9‰ V-SMOW to + 2.8‰ V-SMOW for the birds of prey (Fig. 2A). Uncertainties associated with the drinking water estimates equal 0.50 ± 0.03‰ and correspond to the propagation of uncertainty calculated as the quadratic sum of the analytical uncertainties and the mean standard error of the estimate of the bird phosphate-water fractionation equation (σ_est_ =  ± 0.45)^12^. Only 3 ibises and 3 birds of prey have drinking water δ^18^O_w_ values that overlap the δ^18^O_w_ range of the Nile estimated from Egyptians δ^18^O_p_ values (^39^; Fig. 2A). Carbon isotope compositions of apatite carbonate range from − 16.4 to − 11.63‰ for ibises and from − 17.0 to − 9.1‰ for the birds of prey. The ranges in δ^13^C_c_ values of both ibises (Levene’s test *p* value = 0.028) and birds of prey (Levene’s test *p* value = 0.027) are both significantly larger than that of contemporaneous Egyptians (Fig. 2B). ^87^Sr/^86^Sr values of ibises range from 0.70767 to 0.70901 and from 0.70719 to 0.71994 for the birds of prey. Interestingly, 8 out of 11 ibises have ^87^Sr/^86^Sr values within the range of Nile sediments whereas all birds of prey have ^87^Sr/^86^Sr values out of the range of Nile sediment values. Ibises are clearly separated from birds of prey in their distribution of elemental Sr/Ca ratios, but not of Ba/Ca concentration ratios (Fig. 3).

**Figure 4:**
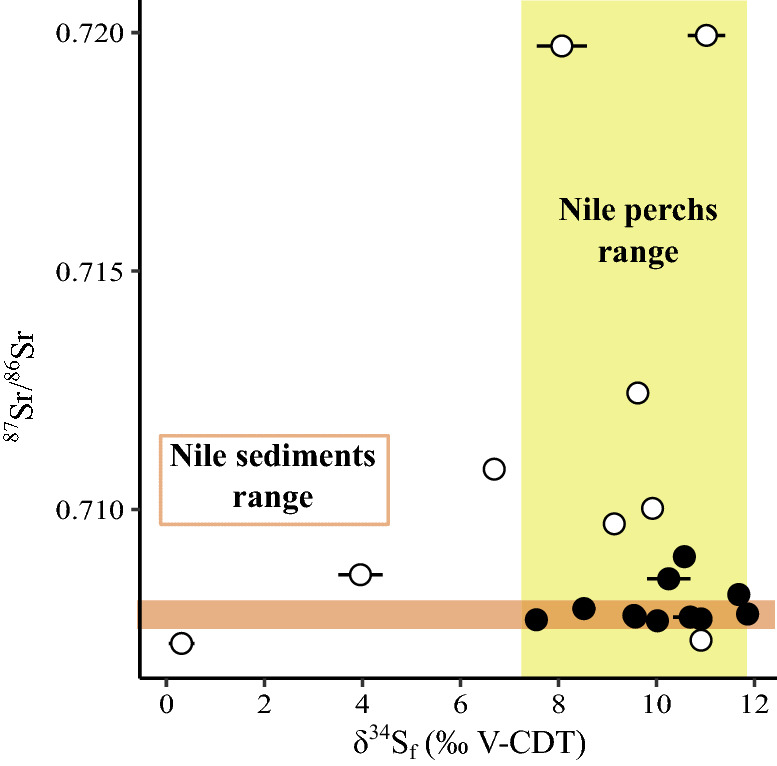
Relationship between bone ^87^Sr/^86^Sr and feather δ^34^S_f_ of the birds. Open circles = birds of prey and filled circles = ibises. Brown range determines the ^87^Sr/^86^Sr range of Nile sediments^37,70^ and yellow band determines the range of δ^34^S values of Nile perches^40^, assumed to reflect the sulfur isotope composition of the Nile environmental sulfur.

**Figure 5:**
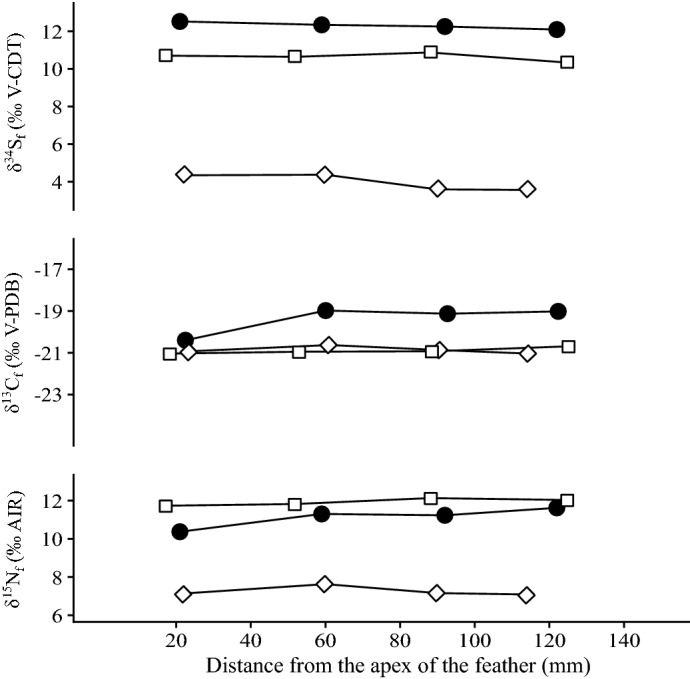
δ^15^N_f_, δ^13^C_f_ and δ^34^S_f_ variations recorded along the growth axis of three whole feathers. Circles correspond to specimen 90001342 (ibis); diamonds to specimen 90010164 (bird of prey) and squares to specimen 90010070 (bird of prey).

Bird feathers have δ^13^C_f_ values ranging from − 21.48 to − 17.13 ‰ for ibises and from − 21.48 to − 19.04 ‰ for birds of prey (Table S2; Fig. 2C). Feather δ^15^N_f_ values range from 10.07 to 13.91 ‰ for ibises and from 7.26 to 14.96 ‰ for birds of prey (Table S1; Fig. 2D). Feather δ^34^S_f_ values range from 7.55 to 11.86 ‰ for ibises and from 0.31 to 11.02 ‰ for birds of prey (Table S2; Fig. 4). Incrementally sampled feathers from the birds of prey specimens 90010164, 90010070 and the ibis specimen 90001342, display isotopic variations along their growth axis of 0.41 ‰, 1.42 ‰ and 0.35 ‰ in δ^13^C_f_, of 0.54 ‰, 1.25 ‰ and 0.40 ‰ δ^15^N_f_ and of 0.81 ‰, 0.43 ‰ and 0.54 ‰ in δ^34^S_f_, respectively (Fig. 5).

## Discussion

### Preservation of the biological stable isotope compositions

**Figure 6:**
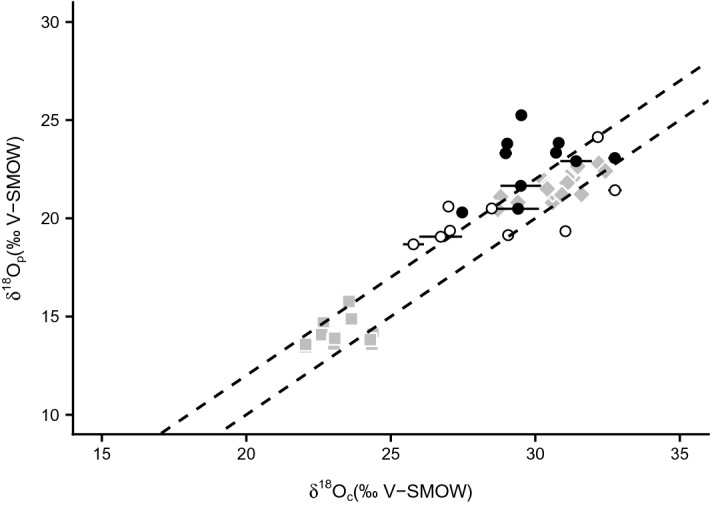
Oxygen isotope compositions of bone phosphate (δ^18^O_p_) reported as a function of the isotopic composition of structural carbonate (δ^18^O_c_) in apatite. Open circles = birds of prey; filled circles = ibises; grey squares = mummified animals from Touzeau et al.^39^; grey diamonds = extant birds from Stanton Thomas and Carlson (2004)^67^ (Table S5). The dashed lines (slope = 1 and intercept = 9 ± 1 ‰) represent the biological phosphate-carbonate fractionation of extant mammals^89^.

Prior to interpreting stable isotope compositions in terms of diet and drinking water sources, primary preservation of the stable isotope compositions needs to be assessed. Indeed, biotic and abiotic processes leading to the decomposition, burial and fossilization of living organisms may alter the original isotopic composition of bioapatite through processes of secondary precipitation, ion adsorption or dissolution–recrystallization of bioapatite^62–66^. In the present case, the process of bird mummification aiming at preserving biological tissues over time from environmental decomposition, and the storage of the mummified birds in catacombs (or tombs) located in the desert environments of Egypt argue for a good preservation of the primary isotopic compositions of bone and feather. Here the pristine nature of the oxygen isotope compositions of bird apatite is indicated by the difference in δ^18^O value between apatite phosphate and carbonate within the range of extant birds^67^, as well as within the range of animal and human mummies published in Touzeau et al.^39^ for which primary preservation of isotopic compositions has been demonstrated (Fig. 6). As diagenetic source, strontium can be leached from soil into bone, an unlikely case given that specimens have not been buried. Moreover, strontium concentration in a study on modern mammal bioapatite averages 360 ppm in carnivore dentine and 538 ppm in herbivore dentine^69^, in the range of studied bird bone values (Table S1) and in agreement with a biopurification process^69^.

In the case of feathers, the percentage of nitrogen, carbon and sulfur in proteins can be indicators of the extent of their alteration. In feathers of extant common blackbird *Turdus merula* and chicken *Gallus gallus*, measured carbon percentage varies from 45.0 to 49.2%, nitrogen varies from 13.1 to 15.9% and sulfur varies from 2.7 to 3.8% (Table S2). These contents match those measured in feathers of mummified birds, with carbon percentage varying from about 45.0 to 53.6%, nitrogen varying from about 12.2 to 15.0% and sulfur varying from about 3.0 to 4.2%, with the exception of specimen 90010069.1 having 7.3% of nitrogen and 1.6% of sulfur. As there is no significant correlation between isotopic composition and percentage of the related element, the stable isotope contamination of feather by the organic balm was most likely negligible. We therefore considered that studied bones and feathers have preserved their original isotope compositions and can be interpreted in terms of the ecology of birds.

### Long-term record of bones

**Figure 7:**
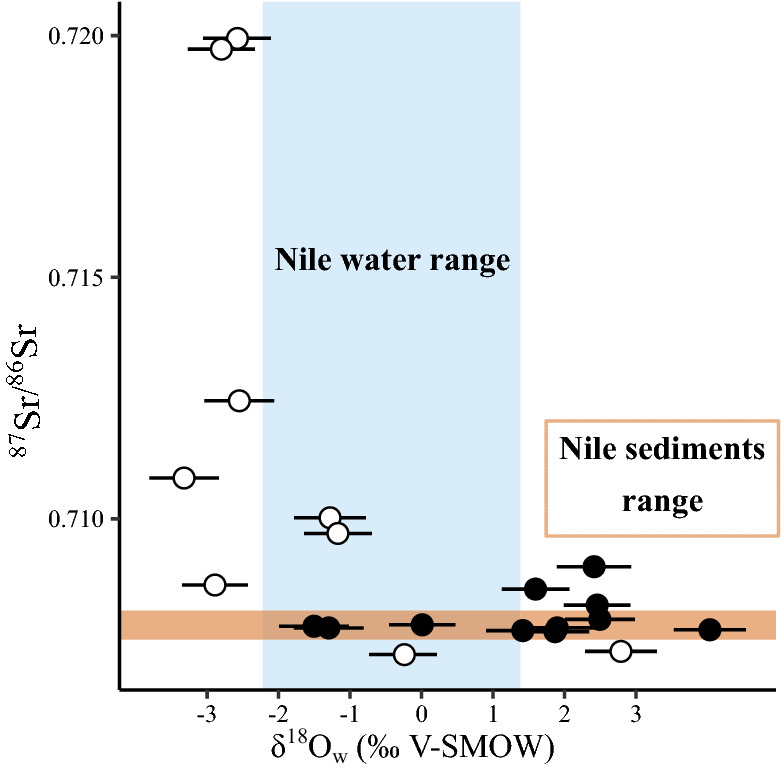
Relationship between the ^87^Sr/^86^Sr of birds and the calculated δ^18^O_w_ of their drinking water. Open circles = birds of prey and filled circles = ibises. The brown band determines values the ^87^Sr/^86^Sr range of Nile sediments^37,70^ and the blue band determines the δ^18^O_w_ range recorded by contemporaneous Egyptians^39^.

According to their combined δ^18^O_p_-derived δ^18^O_w_ and ^87^Sr/^86^Sr values, most birds of prey did not forage in the environments of the Nile valley^37,69^; Fig. 2A, Fig. 7), at least as recorded in the time window of their bone mineralization turnover. Assuming a wild origin for the birds of prey, their exotic ^87^Sr/^86^Sr values and low drinking water δ^18^O_w_ values can be related to their migratory areas in environments of higher latitudes with a more radiogenic substratum. For instance, specimen 90010051 is identified as a Great Spotted eagle (*Clanga clanga*), a species that is today a medium to long-distance migrant breeding within a large forested area ranging from north- eastern Europe eastwards to eastern Asia^71^. Another specimen (90010164) is identified as a Northern Long-legged buzzard (*Buteo rufinus rufinus*), a subspecies that is today a medium to long-distance migrant breeding in the steppes of central Eurasia^72^). The other subspecies, the Atlas Long-legged buzzard *B. r. cirtensis*, is easily recognizable from bone size and proportions, and can be positively excluded here; in contrast to *B. rufinus rufinus*, this subspecies is sedentary in the Atlas Mountains.

Ibises studied here correspond to *Threskiornis aethiopicus*; it is absent in Egypt since the middle of the nineteenth century^73^, and has a distribution area covering a large part of Sub-Saharan Africa^74^). The African Sacred ibis is known to migrate seasonally within the African continent, or to be nomadic, following rains to breed (northward N of equator, southward S of equator), and returning to lower latitudes after the rainy season^74,75^. According to a testimony of Jules-César Savigny and Geoffroy Saint-Hilaire at the turn of the eighteenth and nineteenth centuries, during Bonaparte’s Expedition of Egypt, African Sacred ibises migrated in Egypt and resided there from the end of June to mid-January^76,77^. These authors also mention a previous expedition to the source of the Nile by Bruce, in Ethiopia, in the early eighteenth century, attesting to the arrival of these ibises similarly in late June and departure in mid-January. In the Egyptian Antiquity, African Sacred ibises were known to arrive with the flooding of the Nile^78^, starting indeed end of June. Interestingly, studied mummified ibis bones have strontium isotope signatures of the Nile sediments^37,69^ (Fig. 4), supporting a permanent residency and limited migrations along the course of Nile and its tributaries between Ethiopia, Sudan and Egypt. Moreover, the drinking water of most of them have more positive δ^18^O_w_ value than the average range of the Nile waters (Fig. 7), indicating that they ingested evaporated waters from either breeding ponds (if they grew in captivity), or temporarily isolated and evaporated natural bodies of waters such as oxbow lakes or flood basins. The smaller Glossy ibis, *Plegadis falcinellus*, although known as mummified, and difficult to separate due to the bad state of preservation of mummies, is only very occasional compared to the vast majority of African Sacred ibis, including in the collections concerned by the present study^78^. The isotopic trends observed can therefore confidently be assigned to the African Sacred ibis, at least in most part.

Carbon isotope compositions of bone apatite from both ibises and birds of prey show a variability greater than that of the bones of contemporaneous Egyptians^40^ (Fig. 2B). According to the hypothesis that reared animals would be either fed a rather homogeneous diet (such as food from a dedicated production that was most likely local and rather homogeneous), or fed with some components of the diet of their breeders (such as remains of food production not eaten by Egyptians), they would display a lesser or at least similar isotopic variability than contemporaneous Egyptians. A third possibility is that birds were raised captive in semi-natural enclosures along the Nile valley and would have foraged in these spatially and isotopically limited natural environments. Bone apatite δ^13^C_c_ variability suggests that studied birds fed on more isotopically diversified food resources than Egyptians and were likely wild animals. It is noteworthy that a difference of bone turnover rate and precipitation timing may partly account for the observed differences between birds and humans. Whereas human bones are completely remodeled at a rate of 5–10% per year, averaging an isotopic record of about a decade or more, bird bones is mainly composed of “primary bone” growing during the first year of their life and not remodeled, the remaining bone being “secondary bone” remodeled at a higher rate and leading to its complete renewal within a year^79,80^^.^ It could be argued that the time-averaging difference between human and bird bones results in a higher carbon isotopic variability in birds than humans. However, the same pattern can be observed between bird feather and human hair keratin (see below), supporting different feeding sources between birds and humans.

Moreover, Sr/Ca ratios of bird bones show a clear separation between ibises and birds of prey, the former having bones more concentrated in strontium (Fig. 3). Considering that both bird groups are carnivorous and might have similar Sr assimilation metabolism, this separation would hint to a difference in foraging environment^69,81^, ibises consuming aquatic preys whereas birds of prey consuming more terrestrial ones.

Stable oxygen and carbon isotope compositions as well as ^87^Sr/^86^Sr ratios of bone apatites from the studied mummified birds support a wild origin of the birds of prey. The origin of ibises is less clear as oxygen and strontium isotope composition point to a permanent residence along the course of the Nile, but carbon isotope variability suggests a wild foraging environment that would support the conclusions of Wasef (2019)^11^. According to both their genetic diversity and the exotic origin or large variability of their isotopic compositions, birds used as votive mummies could not have come from a centralized industrial scale farming. However, sustained short-term taming might have been the common practice to satisfy the ritual demand, an hypothesis which can be investigated through the short term record of isotopic compositions of bird feathers.

### Short-term record of feathers

Bulk feather δ^13^C_f_ and δ^15^N_f_ values of both ibises and birds of prey have a large variability that tend to be larger than the variability of Egyptian hair^40^ (Fig. 2C,D). but this observation might be biased by the small number of values for Egyptians (N = 5) compared to those of birds. Studies of moult in precise species of birds of prey are rare, but available data indicate that, including for the Greater Spotted eagle (sample 90010051) and the Northern Long-legged buzzard (sample 90010164), the moult of remiges and retrices in migratory species occurs either: (i) from July to September and again in winter (two-step moult)^82^, or (ii) continuously from July through autumn to winter (many birds of prey including probably *B. r. rufinus*;^82,83^), or (iii) starting earlier in spring through summer to September–October, but not in winter (e.g., the Golden eagle^84^). In all cases, moult therefore occurs through the crossing of very different latitudes, habitats and climates, from spring–summer in breeding areas, through autumn during migration, to winter in wintering grounds. Compared to the δ^34^S_f_ value range of contemporaneous Nile perches^40^ (Fig. 4), the feathers of most birds of prey have a δ^34^S_f_ values within the same range, with the exception of three birds of prey including the Long-legged buzzard (sample 90010164) that have δ^34^S_f_ values indicating that they grew out of the Nile Valley. This variability is consistent with moult occurring at different degrees in the wintering, breeding grounds, or in between. But at least three unambiguous examples reveal wild origin as northern breeders.

Feather growth of ibises extend from March to August^85^. Their δ^34^S_f_ values are within the expected range of the Nile valley (Fig. 4) and confirm altogether with the ^87^Sr/^86^Sr of bones, that ibises were most likely permanent residents along the course of the Nile. As carbon and nitrogen isotope variability and genetic diversity^11^ preclude large scale farming of ibises, the most probable scenario is that wild ibis populations living within the Nile Valley in Egypt were regularly hunted for the demand of votive mummies, as has been observed with fishing and crocodiles hunting^86,87^.

Isotopic variations within the same feather (Fig. 5) are minor and do not indicate any significant change of habitat during their growth. In most case, moult of birds occur before or after migration period because of the significant energetic demand of the migration^88^. Consequently, the absence of intra-feather variation could be explained by the exclusive growth of these one in wintering or breeding area. Stable isotope compositions of feathers do not support short term (less than a season) taming of ibises, suggesting alternatively that hunted birds were killed right away and mummified.

## Conclusion

Stable isotope compositions of oxygen, carbon, strontium, nitrogen and sulfur, along with ^87^Sr/^86^Sr ratios from either bone or feather support a wild origin of mummified ibises and birds of prey. Long and short-term tendencies recorded in bone and feather isotopic compositions indicate that birds of prey migrated outside the Nile Valley, whereas ibises were more permanent residents, moving along the course of the Nile and its tributaries. From the studied sample set, it seems that large scale breeding of birds for mummification was unlikely, as supported by a genomic study^11^, but also short-term taming as evidenced by large stable isotope variability. Birds used as votive mummies might not have been bred, but a dedicated isotopic study on other ones such as sacred mummies and pet mummies would clarify the relationship between humans and birds in ancient Egypt.

## Supplementary information

Supplementary data.

Supplementary Information 1.

Supplementary Information 2.

Supplementary Information 3.

Supplementary Information 4.

Supplementary Information 5.
