# Supplementary material for: Isotopic systematics point to wild origin of mummified birds in Ancient Egypt
Source: Sci Rep. 2020 Sep 22;10:15463. doi: 10.1038/s41598-020-72326-7 (PMC7508811; doi:10.1038/s41598-020-72326-7)
Supplement: Supplementary file 1 — Supplementary data. [file 41598_2020_72326_MOESM1_ESM.pdf]

Isotopic systematics point to wild origin of mummified birds in Ancient Egypt

Marie Linglin[a, g], Romain Amiot[a, *], Pascale Richardin[b], Stéphanie Porcier[c], Ingrid Antheaume[a], Didier Berthet[d], Vincent Grossi[a], François Fourel[e], Jean-Pierre Flandrois[f], Antoine Louchart[a], Jérémy Martin[a], Christophe Lécuyer[a]

[a] UMR 5276, Laboratoire de Géologie de Lyon, Terre, Planètes et Environnement, Université Claude Bernard Lyon 1/CNRS/École Normale Supérieure de Lyon, 69622 Villeurbanne Cedex, France; marie.linglin@ens-lyon.fr; romain.amiot@univ-lyon1.fr; vincent.grossi@univ-lyon1.fr; antoine.louchart@ens-lyon.fr; jeremy.martin@ens-lyon.fr; christophe.lecuyer@univ-lyon1.fr

[b] Centre de Recherche et de Restauration des Musées de France (C2RMF), Palais du Louvre, Porte des Lions, 14 quai François Mitterrand, 75001 Paris, France; UMR 7055, Préhistoire et Technologie (Pretech), Université Paris Nanterre / CNRS, 21 allée de l'Université, 92023 Nanterre Cedex ; pascale.richardin@culture.gouv.fr

[c] Laboratoire CNRS "Histoire et Sources des Mondes Antiques" (HiSoMA-UMR 5189), Maison de l'Orient et de la Méditerranée, Lyon, France; sporcier@hotmail.com

[d] Musée des Confluences, Lyon, France; didier.berthet@museedesconfluences.fr

[e] Laboratoire d'Ecologie des Hydrosystèmes Naturels et Anthropisés, CNRS UMR 5023, Université Claude Bernard Lyon 1, France; francois.fourel@univ-lyon1.fr

[f] Univ Lyon, Université Lyon 1, CNRS, UMR5558, Laboratoire de Biométrie et Biologie Évolutive, 43 bd du 11 novembre 1918, F-69622, Villeurbanne, France; jeanpierreflandrois@icloud.com

[g] Aix Marseille Univ, CNRS, Minist culture, LAMPEA, Aix-en-Provence, France

[*] Corresponding author.

Supplementary data

Feather cleaning procedure test

In order to test the effect of 4 cleaning procedures on the isotopic preservation of feather carbon, nitrogen and sulfur, and choose to most appropriate to remove mummification balms from ibis and birds of prey feathers, 3 feathers of wild Common Blackbird *Turdus merula* and 3 feathers of the Chicken *Gallus gallus* have been collected. The left side of each feathers was only cleaned with distilled water and served as a reference for the true value of the feather. All 5 feathers have been cut longitudinally into 2 parts. Each part was then cut into 5 sections of about 2.5 cm long (**Fig. 1sup**). The two upper sections of the feathers were only cleaned three times with distilled water for 5 minutes in an ultrasonic bath. The comparison of these two sections makes it possible to check the isotopic symmetry of the feather. Then, the 4 sections of the right part underwent 4 different cleaning protocols. One section was cleaned three times with a mixture of dichloromethane / methanol (4: 1) for 5 minutes in an ultrasonic bath. The second section was cleaned twice with a mixture of dichloromethane / methanol (3: 1) for 5 minutes in an ultrasonic bath, then twice with acetone for 5 minutes in an ultrasonic bath. The third section underwent the opposite procedure: twice with acetone and then twice with Dichloromethane / Methanol (3: 1). Finally, the fourth section was cleaned with a dichloromethane / acetone solution (3: 1) three times and with ultrasonic bath. Stable carbon, nitrogen ans sulfur isotope compositions of each treated section was compared to those of the reference section which was symmetrical to it (**Fig. 1S**). The isotopic variations between the treated sections and their reference range up to 0.8‰ for sulfur and carbon, and 0.6‰ for nitrogen. The isotopic variations between the two reference sections being themselves equal to 0.3‰ for sulfur, 0.6‰ for carbon and 0.5‰ for nitrogen (**Supplementary table 3**). Therefore the isotopic differences induced by the treatments can be considered as negligible.

Fig.1S (Below): Isotopic comparison between the treated part (Right) and non treated reference part (Left) of the feathers for sulfur ($\delta^{34}S$), carbon ($\delta^{13}C$) and nitrogen ($\delta^{15}N$). The detail of the different sections with a summary of treatments applied are presented above.

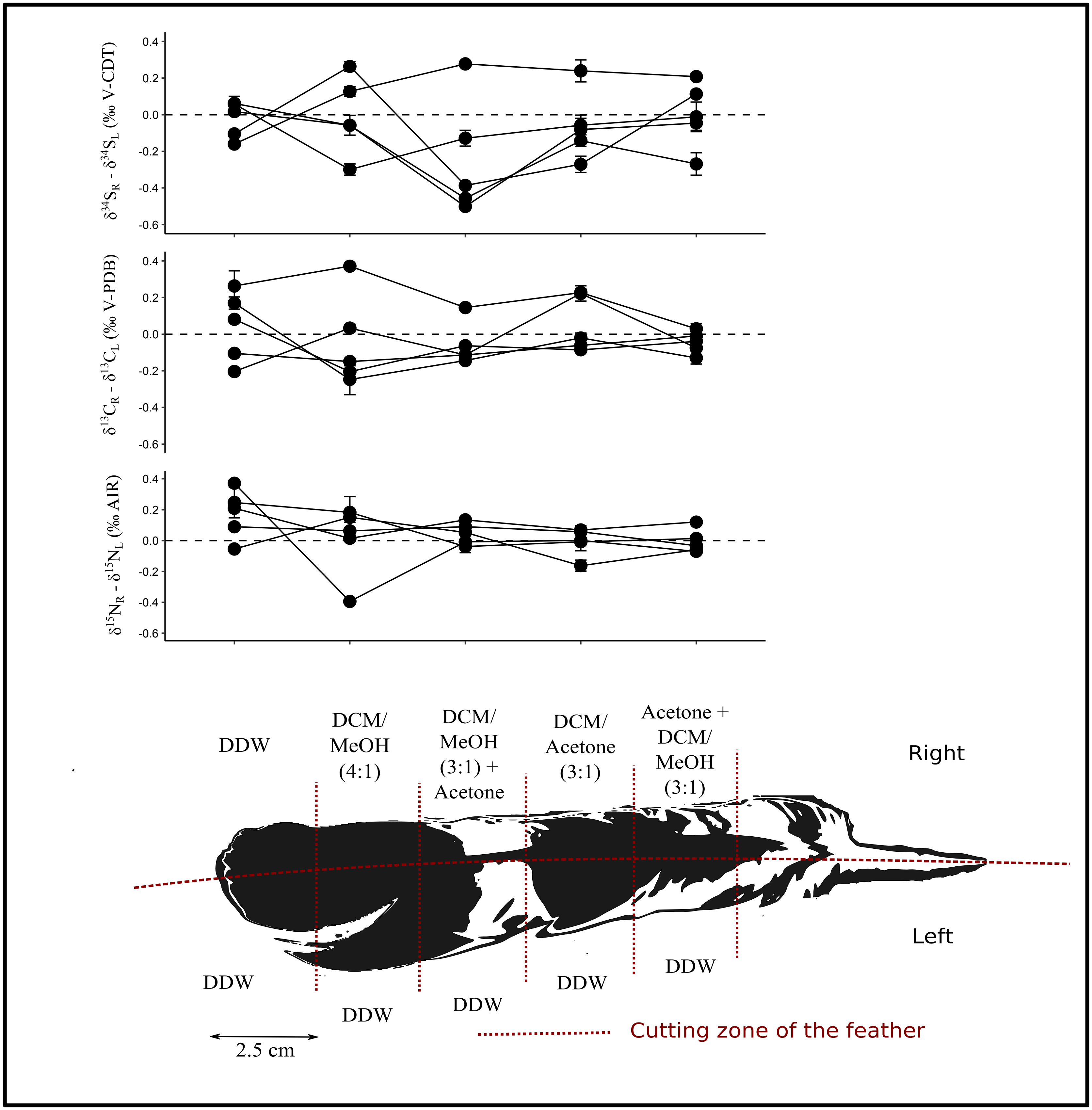
δ34SR - δ34SL (‰ V-CDT)
δ13CR - δ13CL (‰ V-PDB)
δ15NR - δ15NL (‰ AIR)
0.4
0.2
0.0
-0.2
-0.4
-0.6
DDW
DCM/ MeOH (4:1)
DCM/ MeOH (3:1) + Acetone
DCM/ Acetone (3:1)
Acetone + DCM/ MeOH (3:1)
Right
Left
DDW
DDW
DDW
DDW
DDW
2.5 cm
Cutting zone of the feather
