# Supplementary material for: Isotopic systematics point to wild origin of mummified birds in Ancient Egypt
Source: Sci Rep. 2020 Sep 22;10:15463. doi: 10.1038/s41598-020-72326-7 (PMC7508811; doi:10.1038/s41598-020-72326-7)
Supplement: Supplementary file 2 — Supplementary Information 1. [file 41598_2020_72326_MOESM2_ESM.pdf]

Collection #	Sample #	Taxon	Material	Locality	Period	Radiocarbon age (BP)	$\delta^{18}O_p$	(‰ V-SMOW)	$\delta^{18}O_c$	(‰ V-SMOW)	$\delta^{13}C_c$	(‰ V-PDB)		$^{87}Sr/^{86}Sr$	Wt %	CO_3	Ca (%)	Sr (ppm)	Ba (ppm)
							mean	SD	mean	SD	mean	SD	mean	2SD	mean	SD			
90002482	MO1os	Ibis	bone	Egypt	Late Period	2390 ± 30*	20.3	0.2	27.47	-	-11.63	-	0.707778	0.000076	2.7	-	45.2	485.9	72.5
90002491	MO2os	Ibis	bone	Roda	Ptolemaic Period	2010 ± 30*	25.3	0.2	29.52	0.08	-16.42	0.13	0.707705	0.000051	1.2	0.1	41.0	378.0	19.3
90002490	MO3os	Ibis	bone	Egypt	Ptolemaic Period	2095 ± 30	23.9	0.2	-	-	-13.49	0.12	0.707922	0.000046	2.0	0.1	50.6	482.5	29.3
90002489	MO4os	Ibis	bone	Egypt	Ptolemaic Period	2100 ± 30	20.5	0.2	29.40	0.72	-14.15	0.16	0.707744	0.000039	2.2	-	46.2	453.2	21.9
90002498	MO9os	Ibis	bone	Egypt	Ptolemaic Period	2150 ± 40	23.3	0.3	30.72	-	-13.04	-	0.707745	0.000038	2.5	-	46.3	467.9	36.7
90001342	MO10os	Ibis	bone	Egypt	Late Period	2350 ± 30	23.8	0.1	30.81	-	-14.73	-	0.708217	0.000068	1.5	-	36.7	383.4	6.8
90002496	MO11os	Ibis	bone	Egypt	Ptolemaic Period	2245 ± 30	23.8	0.3	29.03	-	-14.05	-	0.709011	0.000045	2.0	-	34.9	974.3	8.3
90002497	MO12os	Ibis	bone	Egypt	Ptolemaic Period	2270 ± 30	23.1	0.1	32.75	0.22	-12.29	0.16	0.708547	0.000060	2.5	0.3	55.9	914.5	56.9
90002492	MO13os	Ibis	bone	Egypt	Ptolemaic Period	2125 ± 30 and 2180 ± 30	22.9	0.2	31.41	0.54	-12.22	0.07	0.707688	0.000074	1.7	0.0	43.5	537.3	38.0
90002493	MO14os	Ibis	bone	Egypt	Ptolemaic Period	2070 ± 30	21.7	0.1	29.50	0.70	-14.43	0.24	0.707809	0.000069	0.9	0.3	28.3	264.8	25.1
90002494	MO15os	Ibis	bone	Egypt	Ptolemaic Period	2155 ± 30	23.3	0.2	28.98	0.21	-16.40	0.05	0.707666	0.000055	1.4	0.0	40.6	558.4	38.5
90010164	MO5os	*Buteo rufinus rufinus*	bone	Luxor	Ptolemaic Period	2160 ± 50*	19.1	0.1	26.73	0.74	-15.84	0.23	0.708632	0.000137	1.5	0.1	26.9	153.4	16.6
90010165	MO6os	Bird of prey	bone	Egypt	Roman Period	1915 ± 30	21.4	0.2	32.76	0.23	-13.51	0.03	0.707191	0.000084	2.0	0.2	49.0	230.5	18.6
90010166	MO7os	Bird of prey	bone	Egypt	Roman Period	1925 ± 30	19.3	0.2	31.04	0.14	-15.09	0.05	0.719941	0.000109	1.4	0.3	39.9	103.8	120.3
90010167	MO8os	Bird of prey	bone	Egypt	Roman Period	1935 ± 30	19.1	0.1	29.06	0.12	-9.12	0.17	0.719720	0.000095	2.6	0.5	46.2	265.5	123.9
90010070	MO16os	Bird of prey	bone	Egypt	Roman Period	1870 ± 30	24.1	0.2	32.16	0.11	-17.00	0.14	0.707259	0.000075	1.9	0.5	44.1	232.8	19.6
90010069.1	MO17os	Bird of prey	bone	Egypt	Ptolemaic Period	2195 ± 30	18.7	0.2	25.78	0.36	-13.10	0.27	0.710846	0.000099	1.1	0.1	46.7	122.1	27.3
90010069.2	MO18os	Bird of prey	bone	Egypt	Roman Period	1835 ± 30	20.5	0.2	28.49	0.13	-12.86	0.03	0.710023	0.000129	1.6	0.3	42.7	256.1	45.7
90010051	MO19os	*Clanga clanga*	bone	Giza	Roman Period	1915 ± 30	19.4	0.2	27.05	-	-15.36	-	0.712443	0.000100	2.1	-	42.4	147.9	20.3
90010054	MO20os	Bird of prey	bone	Kôm Ombo	Ptolemaic Period	2030 ± 30	20.6	0.1	26.99	0.03	-16.86	0.03	0.709699	0.000098	1.5	0.4	41.3	141.9	38.8

Table S1: Oxygen and carbon isotope compositions of apatite carbonate ($\delta^{18}O_c$ and $\delta^{13}C_c$) and phosphate ($\delta^{18}O_p$) of apatite from mummified birds are reported along with their $^{87}Sr/^{86}Sr$ ratios and their relative abundances in strontium (Sr), barium (Ba) and calcium (Ca). * specimen dating published in Richardin et al. (2017)

Reference:

Richardin, P., Porcier, S., Ikram, S., Louarn, G. & Berthet, D. Cats, Crocodiles, Cattle, and More: Initial Steps Toward Establishing a Chronology of Ancient Egyptian Animal Mummies. Radiocarbon 59, 595–607 (2017).
