# Supplementary material for: Isotopic systematics point to wild origin of mummified birds in Ancient Egypt
Source: Sci Rep. 2020 Sep 22;10:15463. doi: 10.1038/s41598-020-72326-7 (PMC7508811; doi:10.1038/s41598-020-72326-7)
Supplement: Supplementary file 3 — Supplementary Information 2. [file 41598_2020_72326_MOESM3_ESM.pdf]

Collection #	Sample #	Taxon	Material	Locality	Period	Radiocarbon age (BP)	$\delta^{15}N$ mean	(‰ AIR) SD	N mean	(%) SD	$\delta^{13}C$ mean	(‰ V-PDB) SD	C mean	(%) SD	$\delta^{34}S$ mean	(‰ V-CDT) SD	S mean	(%) SD	C/N	C/S
90002482	MO1pl	Ibis	feather fragment	Egypt	Late Period	2390 ± 30	11.88	0.15	13.89	0.14	−21,48	0.14	48.61	0.07	9.54	0.14	2.96	0.04	3.50	16.40
90002491	MO2pl	Ibis	feather fragment	Roda	Ptolemaic Period	2010 ± 30	13.24	0.14	14.11	0.02	−20,35	0.02	51.25	0.22	10.91	0.18	3.45	0.10	3.60	14.90
90002490	MO3pl	Ibis	feather fragment	Egypt	Ptolemaic Period	2095 ± 30	13.36	0.17	13.94	0.03	−18,85	0.25	50.64	0.30	8.52	0.24	3.23	0.03	3.60	15.70
90002489	MO4pl	Ibis	feather fragment	Egypt	Ptolemaic Period	2100 ± 30	12.71	0.11	12.72	0.04	−19,15	0.16	44.99	0.05	10.69	0.35	4.19	0.27	3.50	10.70
90002498	MO9pl	Ibis	feather fragment	Egypt	Ptolemaic Period	2150 ± 40	10.13	0.25	14.25	0.30	−19,20	0.10	49.78	0.34	9.57	0.17	3.51	0.13	3.50	14.20
90001342	MO10pl	Ibis	feather fragment	Egypt	Late Period	2350 ± 30	11.07	0.03	14.48	0.30	−19,00	0.15	48.91	0.28	11.68	0.11	3.37	0.09	3.40	14.50
90002496	MO11pl	Ibis	feather fragment	Egypt	Ptolemaic Period	2245 ± 30	13.91	0.05	14.31	0.12	−19,61	0.08	49.57	0.03	10.57	0.20	3.51	0.15	3.50	14.10
90002497	MO12pl	Ibis	feather fragment	Egypt	Ptolemaic Period	2270 ± 30	11.44	0.03	14.09	0.05	−19,35	0.13	50.31	0.19	10.25	0.44	3.83	0.07	3.60	13.10
90002492	MO13pl	Ibis	feather fragment	Egypt	Ptolemaic Period	2125 ± 30 and 2180 ± 30	11.27	0.21	14.99	0.24	−19,55	0.26	51.44	0.03	7.55	0.10	3.42	0.21	3.40	15.00
90002493	MO14pl	Ibis	feather fragment	Egypt	Ptolemaic Period	2070 ± 30	10.07	0.20	14.81	0.16	−17,13	0.86	49.68	0.17	11.86	0.02	4.07	0.07	3.40	12.20
90002494	MO15pl	Ibis	feather fragment	Egypt	Ptolemaic Period	2155 ± 30	13.52	0.02	12.20	0.06	−21,45	0.06	47.39	0.24	10.02	0.16	4.11	0.12	3.90	11.50
90010164	MO5pl	*Buteo rufinus rufinus*	feather fragment	Luxor	Ptolemaic Period	2160 ± 50	7.26	0.17	14.99	0.62	−20,87	0.22	51.55	0.43	3.96	0.45	3.34	0.14	3.45	14.05
90010165	MO6pl	Bird of prey	feather fragment	Egypt	Roman Period	1915 ± 30	14.96	0.11	14.86	0.11	−19,83	0.09	49.27	0.09	0.31	0.26	3.77	0.06	3.30	13.10
90010166	MO7pl	Bird of prey	feather fragment	Egypt	Roman Period	1925 ± 30	9.44	1.08	14.71	0.10	−21,48	0.28	51.19	1.06	11.02	0.38	3.47	0.10	3.50	14.80
90010167	MO8pl	Bird of prey	feather fragment	Egypt	Roman Period	1935 ± 30	11.28	0.21	15.22	0.13	−19,04	0.63	50.99	0.25	8.07	0.51	3.52	0.17	3.30	14.50
90010070	MO16pl	Bird of prey	feather fragment	Egypt	Roman Period	1870 ± 30	11.63	0.11	14.05	0.14	−21,12	0.05	51.84	0.19	10.91	0.03	3.76	0.16	3.70	13.80
90010069.1	MO17pl	Bird of prey	feather fragment	Egypt	Ptolemaic Period	2195 ± 30	14.91	0.23	7.24	0.09	−21,05	0.08	51.97	0.18	6.69	0.13	1.64	0.13	7.20	31.60
90010069.2	MO18pl	Bird of prey	feather fragment	Egypt	Roman Period	1835 ± 30	12.04	0.02	14.46	0.27	−20,69	0.02	53.58	0.11	9.92	0.22	3.47	0.11	3.70	15.40
90010051	MO19pl	*Clanga clanga*	feather fragment	Giza	Roman Period	1915 ± 30	8.64	0.08	14.17	0.22	−20,98	0.00	48.64	0.21	9.62	0.13	3.70	0.16	3.40	13.10
90010054	MO20pl	Bird of prey	feather fragment	Kôm Ombo	Ptolemaic Period	2030 ± 30	9.83	0.14	14.79	0.19	−20,54	0.11	51.00	0.33	9.14	0.08	3.51	0.25	3.40	14.50
90010164	MO5pl – 1	*Buteo rufinus rufinus*	feather increment	Luxor	Ptolemaic Period	2160 ± 50	7.14	0.07	15.11	0.18	−20,93	0.21	51.50	0.13	4.34	0.38	3.64	0.10	3.40	14.20
90010164	MO5pl – 2	*Buteo rufinus rufinus*	feather increment	Luxor	Ptolemaic Period	2160 ± 50	7.63	0.07	14.92	0.29	−20,63	0.01	51.31	0.21	4.37	0.06	3.34	0.05	3.40	15.40
90010164	MO5pl – 3	*Buteo rufinus rufinus*	feather increment	Luxor	Ptolemaic Period	2160 ± 50	7.16	0.03	14.98	0.17	−20,87	0.03	51.35	0.28	3.59	0.21	3.19	0.04	3.40	16.10
90010164	MO5pl – 4	*Buteo rufinus rufinus*	feather increment	Luxor	Ptolemaic Period	2160 ± 50	7.09	0.13	14.96	0.5	−21,04	0.07	52.04	0.22	3.56	0.10	3.21	0.07	3.50	16.20
90001342	MO10pl – 1	Ibis	feather increment	Egypt	Late Period	2350 ± 30	10.37	0.15	16	0.05	−20,40	0.18	51.78	0.28	12.52	0.23	3.48	0.03	3.20	14.90
90001342	MO10pl – 2	Ibis	feather increment	Egypt	Late Period	2350 ± 30	11.3	0.08	15.77	0.1	−18,98	0.03	50.78	0.26	12.34	0.06	3.53	0.10	3.20	14.40
90001342	MO10pl – 3	Ibis	feather increment	Egypt	Late Period	2350 ± 30	11.23	0.11	15.95	0.09	−19,13	0.11	50.92	0.30	12.25	0.15	3.48	0.07	3.20	14.70
90001342	MO10pl – 4	Ibis	feather increment	Egypt	Late Period	2350 ± 30	11.62	0.06	15.85	0.43	−19,02	0.23	51.30	0.47	12.09	0.07	3.57	0.16	3.20	14.40
90001342	MO10pl – 5	Ibis	feather increment	Egypt	Late Period	2350 ± 30	11.32	0.07	16.44	0.14	−19,25	0.03	52.37	0.14	12.33	0.01	3.39	0.04	3.20	15.40
90010070	MO16pl – 1	Bird of prey	feather increment	Egypt	Roman Period	1870 ± 30	11.73	0.07	14.31	0.17	−21,04	0.03	47.34	0.56	10.70	0.11	3.57	0.08	3.30	13.30
90010070	MO16pl – 2	Bird of prey	feather increment	Egypt	Roman Period	1870 ± 30	11.82	0	15.63	0.13	−20,95	0.09	48.61	1.56	10.64	0.20	3.39	0.28	3.10	14.30
90010070	MO16pl – 3	Bird of prey	feather increment	Egypt	Roman Period	1870 ± 30	12.13	0.13	15.07	0.02	−20,93	0.10	49.64	0.84	10.87	0.27	3.40	0.05	3.30	14.60
90010070	MO16pl – 4	Bird of prey	feather increment	Egypt	Roman Period	1870 ± 30	12.03	0.11	14.79	0.02	−20,69	0.03	50.82	0.10	10.33	0.09	3.49	0.01	3.40	14.60

Table 2: Nitrogen ($\delta^{15}N_f$), carbon ($\delta^{13}C_f$) and sulfur ($\delta^{34}S_f$) isotope compositions of feather fragments and feather increments of mummified birds along with the relative abundance of C, N and S.
