# Supplementary material for: Isotopic systematics point to wild origin of mummified birds in Ancient Egypt
Source: Sci Rep. 2020 Sep 22;10:15463. doi: 10.1038/s41598-020-72326-7 (PMC7508811; doi:10.1038/s41598-020-72326-7)
Supplement: Supplementary file 4 — Supplementary Information 3. [file 41598_2020_72326_MOESM4_ESM.pdf]

Sample #	Taxon	Material	Locality	Période	chemical cleaning	S	(%)	N	(%)	C	(%)	$\delta^{34}S$	(‰ V-CDT)	$\delta^{15}N$	(‰ AIR)	$\delta^{13}C$	(‰ V-PDB)
						mean	SD	mean	SD	mean	SD	mean	SD	mean	SD	mean	SD
P11left	*Turdus merula*	feather increment left	Lyon	Present-day	DDW	2.87	0.04	13.54	0.62	46.59	1.47	2.84	0.09	4.36	0.01	-24.63	0.09
P11right	*Turdus merula*	feather increment right	Lyon	Present-day	DDW	2.98	0.07	14.07	0.80	47.01	0.91	2.68	0.05	4.31	0.04	-24.37	0.27
P12left	*Turdus merula*	feather increment left	Lyon	Present-day	DDW	2.94	0.14	13.11	1.06	45.03	3.97	2.99	0.09	4.57	0.08	-24.72	0.15
P12right	*Turdus merula*	feather increment right	Lyon	Present-day	DCM/MeOH (4:1)	3.22	0.04	14.88	0.19	47.86	0.49	3.12	0.13	4.72	0.16	-24.35	0.11
P13left	*Turdus merula*	feather increment left	Lyon	Present-day	DDW	3.37	0.02	15.44	0.60	49.22	1.71	3.42	0.04	4.87	0.02	-24.05	0.05
P13right	*Turdus merula*	feather increment right	Lyon	Present-day	DCM/MeOH (3:1) + acetone	3.27	0.04	15.68	0.16	48.46	0.50	3.70	0.11	4.92	0.05	-23.90	0.10
P14left	*Turdus merula*	feather increment left	Lyon	Present-day	DDW	3.50	0.16	14.97	0.53	48.03	1.79	3.35	0.07	4.97	0.12	-23.87	0.08
P14right	*Turdus merula*	feather increment right	Lyon	Present-day	DCM/Acetone (3:1)	3.11	0.31	15.09	0.80	46.80	2.21	3.59	0.24	4.81	0.14	-23.64	0.11
P15left	*Turdus merula*	feather increment left	Lyon	Present-day	DDW	2.86	0.16	15.62	0.29	49.02	0.82	3.81	0.05	4.85	0.04	-23.13	0.13
P15right	*Turdus merula*	feather increment right	Lyon	Present-day	Acetone + DCM/MeOH (3 :1)	2.84	0.17	15.59	0.25	48.64	0.61	4.02	0.08	4.79	0.16	-23.10	0.11
P21left	*Turdus merula*	feather increment left	Lyon	Present-day	DDW	3.44	0.28	15.21	0.09	47.87	0.37	5.38	0.15	3.64	0.29	-24.22	0.17
P21right	*Turdus merula*	feather increment right	Lyon	Present-day	DDW	3.20	0.52	15.11	0.28	49.28	0.79	5.44	0.14	3.88	0.12	-24.05	0.06
P22left	*Turdus merula*	feather increment left	Lyon	Present-day	DDW	3.21	0.18	15.30	0.39	48.08	0.20	5.65	0.16	2.71	0.20	-24.50	0.29
P22right	*Turdus merula*	feather increment right	Lyon	Present-day	DCM/MeOH (4:1)	3.71	0.08	15.48	0.37	48.21	0.67	5.35	0.08	2.90	0.25	-24.75	0.02
P23left	*Turdus merula*	feather increment left	Lyon	Present-day	DDW	3.02	0.10	15.78	0.08	49.24	0.13	5.98	0.18	2.49	0.13	-24.67	0.05
P23right	*Turdus merula*	feather increment right	Lyon	Present-day	DCM/MeOH (:1) + acetone	3.05	0.16	15.56	0.19	47.95	0.48	5.86	0.10	2.45	0.15	-24.82	0.06
P24left	*Turdus merula*	feather increment left	Lyon	Present-day	DDW	2.90	0.08	15.77	0.25	48.80	0.59	6.45	0.13	2.95	0.10	-24.84	0.13
P24right	*Turdus merula*	feather increment right	Lyon	Present-day	DCM/Acetone (3:1)	3.12	0.23	15.85	0.27	49.09	0.92	6.39	0.14	2.94	0.10	-24.86	0.09
P25left	*Turdus merula*	feather increment left	Lyon	Present-day	DDW	3.03	0.13	15.92	0.22	49.29	0.35	6.46	0.08	3.11	0.08	-24.93	0.18
P25right	*Turdus merula*	feather increment right	Lyon	Present-day	Acetone + DCM/MeOH (3 :1)	3.02	0.10	15.57	0.06	47.93	0.15	6.45	0.27	3.13	0.08	-25.06	0.04
P31left	*Turdus merula*	feather increment left	Lyon	Present-day	DDW	2.71	0.01	14.35	0.56	48.47	0.21	5.06	0.09	4.52	0.14	-24.96	0.13
P31right	*Turdus merula*	feather increment right	Lyon	Present-day	DDW	3.23	0.02	14.06	0.06	48.54	0.60	4.95	0.06	4.89	0.03	-25.17	0.00
P32left	*Turdus merula*	feather increment left	Lyon	Present-day	DDW	3.25	0.03	14.38	0.16	47.65	0.35	4.72	0.06	4.76	0.03	-25.05	0.07
P32right	*Turdus merula*	feather increment right	Lyon	Present-day	DCM/MeOH (4:1)	2.79	0.15	15.15	0.37	48.08	0.04	4.98	0.15	4.36	0.13	-25.01	0.12
P33left	*Turdus merula*	feather increment left	Lyon	Present-day	DDW	3.03	0.07	15.07	0.14	48.53	0.20	5.29	0.05	4.65	0.06	-24.69	0.04
P33right	*Turdus merula*	feather increment right	Lyon	Present-day	DCM/MeOH (3:1) + acetone	3.07	0.13	15.35	0.30	48.48	0.37	4.90	0.11	4.64	0.06	-24.80	0.06
P34left	*Turdus merula*	feather increment left	Lyon	Present-day	DDW	2.86	0.31	15.63	0.12	48.78	0.05	5.12	0.19	5.07	0.18	-25.21	0.12
P34right	*Turdus merula*	feather increment right	Lyon	Present-day	DCM/Acetone (3:1)	3.00	0.18	15.63	0.14	48.26	0.17	4.85	0.08	5.07	0.19	-24.98	0.17
P35left	*Turdus merula*	feather increment left	Lyon	Present-day	DDW	2.83	0.20	15.53	0.25	48.74	0.01	4.86	0.13	5.60	0.06	-24.78	0.20
P35right	*Turdus merula*	feather increment right	Lyon	Present-day	Acetone + DCM/MeOH (3 :1)	2.71	0.12	15.64	0.24	48.52	0.27	4.98	0.00	5.53	0.09	-24.86	0.07
P41left	*Gallus gallus*	feather increment left	Lyon	Present-day	DDW	3.63	0.18	15.17	0.11	46.10	0.02	6.09	0.07	3.58	0.07	-21.23	0.06
P41right	*Gallus gallus*	feather increment right	Lyon	Present-day	DDW	3.82	0.03	15.45	0.14	47.49	0.30	6.15	0.02	3.79	0.00	-21.15	0.04
P42left	*Gallus gallus*	feather increment left	Lyon	Present-day	DDW	3.66	0.19	15.43	0.10	47.54	0.20	6.48	0.09	3.65	0.04	-21.30	0.09
P42right	*Gallus gallus*	feather increment right	Lyon	Present-day	DCM/MeOH (4:1)	3.49	0.20	15.23	0.09	46.31	0.32	6.42	0.04	3.67	0.04	-21.50	0.15
P43left	*Gallus gallus*	feather increment left	Lyon	Present-day	DDW	3.05	0.14	15.04	0.25	45.75	0.06	7.69	0.14	3.35	0.12	-21.68	0.13
P43right	*Gallus gallus*	feather increment right	Lyon	Present-day	DCM/MeOH (3:1) + acetone	3.28	0.14	14.95	0.33	46.35	0.36	7.23	0.04	3.49	0.05	-21.74	0.04
P44left	*Gallus gallus*	feather increment left	Lyon	Present-day	DDW	3.16	0.14	15.38	0.07	47.29	0.20	7.33	0.08	3.30	0.04	-21.84	0.06
P44right	*Gallus gallus*	feather increment right	Lyon	Present-day	DCM/Acetone (3:1)	3.22	0.17	15.29	0.24	46.69	0.04	7.18	0.16	3.37	0.16	-21.93	0.05
P45left	*Gallus gallus*	feather increment left	Lyon	Present-day	DDW	3.13	0.03	15.56	0.12	47.89	0.30	6.85	0.07	3.16	0.06	-21.94	0.01
P45right	*Gallus gallus*	feather increment right	Lyon	Present-day	Acetone + DCM/MeOH (3 :1)	3.11	0.08	15.26	0.18	47.47	0.21	6.58	0.24	3.28	0.04	-21.98	0.04
P51left	*Gallus gallus*	feather increment left	Lyon	Present-day	DDW	3.70	0.18	15.42	0.04	47.32	0.25	6.03	0.05	3.68	0.04	-21.08	0.02
P51right	*Gallus gallus*	feather increment right	Lyon	Present-day	DDW	3.74	0.10	15.30	0.03	47.15	0.19	6.05	0.06	3.77	0.04	-21.18	0.04
P52left	*Gallus gallus*	feather increment left	Lyon	Present-day	DDW	3.12	0.04	15.47	0.22	47.69	0.20	6.27	0.22	3.68	0.09	-21.15	0.02
P52right	*Gallus gallus*	feather increment right	Lyon	Present-day	DCM/MeOH (4:1)	3.22	0.12	15.42	0.05	47.53	0.10	6.21	0.08	3.75	0.10	-21.30	0.08
P53left	*Gallus gallus*	feather increment left	Lyon	Present-day	DDW	3.12	0.07	15.62	0.20	48.01	0.57	6.39	0.32	3.94	0.01	-21.20	0.04
P53right	*Gallus gallus*	feather increment right	Lyon	Present-day	DCM/MeOH (3:1) + acetone	3.33	0.15	15.70	0.06	48.44	0.38	5.89	0.08	4.03	0.02	-21.31	0.04
P54left	*Gallus gallus*	feather increment left	Lyon	Present-day	DDW	3.15	0.06	15.64	0.07	48.12	0.23	6.47	0.22	4.13	0.02	-21.45	0.05
P54right	*Gallus gallus*	feather increment right	Lyon	Present-day	DCM/Acetone (3:1)	3.11	0.18	15.58	0.12	47.61	0.21	6.38	0.17	4.19	0.03	-21.51	0.04
P55left	*Gallus gallus*	feather increment left	Lyon	Present-day	DDW	3.13	0.08	15.73	0.23	48.06	0.12	6.37	0.08	4.33	0.04	-21.49	0.09
P55right	*Gallus gallus*	feather increment right	Lyon	Present-day	Acetone + DCM/MeOH (3 :1)	3.18	0.25	15.68	0.36	47.81	0.32	6.33	0.18	4.30	0.11	-21.50	0.01
-	-	chicken food	Lyon	Present-day		0.85	0.04	3.21	0.20	38.41	0.04	4.34	0.27	3.70	0.43	-22.14	0.59

Table 3: Nitrogen ($\delta^{15}N_f$), carbon ($\delta^{13}C_f$) and sulfur ($\delta^{34}S_f$) isotope compositions of feather increments of extant birds along with the relative abundance of C, N and S.
