# Supplementary material for: Isotopic systematics point to wild origin of mummified birds in Ancient Egypt
Source: Sci Rep. 2020 Sep 22;10:15463. doi: 10.1038/s41598-020-72326-7 (PMC7508811; doi:10.1038/s41598-020-72326-7)
Supplement: Supplementary file 5 — Supplementary Information 4. [file 41598_2020_72326_MOESM5_ESM.pdf]

Collection #	Taxon	Material	Locality	Period	$\delta^{15}N_{Keratin}$ (‰ AIR)	$\delta^{13}C_{Keratin}$ (‰ V-PDB)	$\delta^{34}S_{Keratin}$ (‰ V-CDT)	N %	C %	S %	Origin
30000103	Human	Hair	Gournah	26th Dynasty	12.8	-19.2	8.9	15.0	49.6	4.9	Touzeau et al. (2014)
30000257	Human	Hair	Gournah	26th Dynasty	14.2	-19.9	8.1	14.4	49.6	4.5	Touzeau et al. (2014)
30000127	Human	Hair	Thebes	26th Dynasty	15.5	-19.5	7.1	14.8	46.7	5.1	Touzeau et al. (2014)
30000102	Human	Hair	Thebes	26th Dynasty	9.8	-20.2	8.7	14.6	47.8	5.0	Touzeau et al. (2014)
30000125	Human	Hair	Thebes	26th Dynasty	12.1	-20.0	7.8	14.6	47.9	5.0	Touzeau et al. (2014)
90002164.90002659	Nile perch	Scales	-	Greco-Roman	8.8	-19.0	7.2	7.7	28.8	0.9	Touzeau et al. (2014)
90002168.90002260	Nile perch	Scales	-	Greco-Roman	11.3	-18.6	9.8	5.2	25.1	0.9	Touzeau et al. (2014)
90002180.90002225.90002193	Nile perch	Scales	-	Greco-Roman	10.4	-18.9	10.2	5.6	25.2	0.6	Touzeau et al. (2014)
90002196.90002258	Nile perch	Scales	-	Greco-Roman	11.1	-19.0	9.0	7.9	28.7	0.7	Touzeau et al. (2014)
90002231.90002244	Nile perch	Scales	-	Greco-Roman	11.2	-19.4	10.7	4.7	22.8	0.7	Touzeau et al. (2014)
90002248.90002241	Nile perch	Scales	-	Greco-Roman	7.8	-15.1	8.9	8.2	25.6	0.7	Touzeau et al. (2014)
90001349.90002241.90002269	Nile perch	Scales	-	Greco-Roman	11.4	-18.5	10.7	6.0	22.3	0.7	Touzeau et al. (2014)
90002232	Nile perch	Scales	-	Greco-Roman	11.1	-19.1	9.1	4.5	17.0	0.5	Touzeau et al. (2014)
90002238	Nile perch	Large scale	-	Greco-Roman	10.9	-19.9	9.3	6.8	25.8	0.7	Touzeau et al. (2014)
90002167	Nile perch	Scales	-	Greco-Roman	11.3	-19.6	10.6	6.5	27.9	0.7	Touzeau et al. (2014)
90001179	Nile perch	Large scale	Esna	Greco-Roman	9.5	-27.6	11.8	6.9	32.7	0.5	Touzeau et al. (2014)
90002356	Cat	Hair	-	Late Period – Ptolemaic	13.0	-20.6	8.8	14.4	48.3	3.3	Touzeau et al. (2014)
90002644	Rodent	Hair	Kom Ombo	Greco-Roman	4.3	-22.1	9.7	12.9	43.5	3.6	Touzeau et al. (2014)
90001211	Dorcas gazelle	Hair	Kom Mereh	Greco-Roman	12.3	-21.4	7.6	8.8	32.6	1.6	Touzeau et al. (2014)
90010003	Gazelle	Hair	Kom Mereh	Greco-Roman	13.6	-17.3	14.6	14.0	48.1	2.7	Touzeau et al. (2014)
90001404	Dog	Hair	Asyut	Greco-Roman	13.3	-19.7	7.7	15.2	48.8	4.2	Touzeau et al. (2014)
90001403	Dog	Hair	Asyut	Greco-Roman	13.4	-20.7	10.1	14.3	45.1	4.4	Touzeau et al. (2014)
90002324	Dog	Hair	Asyut	Greco-Roman	12.1	-19.7	8.5	14.1	44.2	4.0	Touzeau et al. (2014)
90010094	Bird of preyA	Feather	Gizeh	Late Period – Ptolemaic	12.8	-19.0	11.1	11.7	50.8	2.3	Touzeau et al. (2014)
90010094b	Bird of preyA	Feather	Gizeh	Late Period – Ptolemaic	12.5	-17.2	12.7	14.5	48.0	2.3	Touzeau et al. (2014)

Table S4: published nitrogen ($\delta^{15}N$), carbon ($\delta^{13}C$) and sulfur ($\delta^{34}S$) isotope compositions of feather, hair and scales of mummified birds, fish, humans and other mammals.

Reference:

Touzeau, A. et al. Diet of ancient Egyptians inferred from stable isotope systematics. Journal of Archaeological Science 46, 114–124 (2014).
