# Supplementary material for: Isotopic systematics point to wild origin of mummified birds in Ancient Egypt
Source: Sci Rep. 2020 Sep 22;10:15463. doi: 10.1038/s41598-020-72326-7 (PMC7508811; doi:10.1038/s41598-020-72326-7)
Supplement: Supplementary file 6 — Supplementary Information 5. [file 41598_2020_72326_MOESM6_ESM.pdf]

Collection #	Taxon	Material	Locality	Period	$\delta^{18}O_p$ (‰ V-SMOW)	$\delta^{18}O_c$ (‰ V-SMOW)	$\delta^{13}C_c$ (‰ V-PDB)	Origin
90010250	Fish	bone	Esna	Greco-Roman	21.5	-	-	Touzeau et al. (2013)
90010250	Fish	bone	Esna	Greco-Roman	21.4	-	-	Touzeau et al. (2013)
90010266	Cat	bone	Stabl-Antar	Greco-Roman	21.1	28.8	-15.4	Touzeau et al. (2013)
90010267	Cat	bone	Stabl-Antar	Greco-Roman	20.8	29.4	-15.2	Touzeau et al. (2013)
30000162	Human	bone	Gournah	Greco-Roman	22.8	32.2	-14.4	Touzeau et al. (2013)
30000163	Human	bone	Gournah	Greco-Roman	21.9	30.3	-14.8	Touzeau et al. (2013)
30000164	Human	bone	Gournah	Greco-Roman	22.1	31.3	-14.7	Touzeau et al. (2013)
30000172	Human	bone	Gournah	Greco-Roman	20.5	28.7	-13.5	Touzeau et al. (2013)
30000139	Human	bone	Thebes	Ptolemaic	22.4	31.3	-14.4	Touzeau et al. (2013)
30000161	Human	bone	Thebes	Ptolemaic	21.6	30.4	-14.0	Touzeau et al. (2013)
30000118	Human	bone	Gournah	Ptolemaic	20.8	30.6	-13.9	Touzeau et al. (2013)
30000169	Human	bone	Gournah	Ptolemaic	21.2	31.6	-13.3	Touzeau et al. (2013)
30000196	Human	bone	Gournah	Ptolemaic	21.8	31.1	-13.6	Touzeau et al. (2013)
30000197	Human	bone	Gournah	Ptolemaic	21.3	30.6	-14.5	Touzeau et al. (2013)
30000156	Human	bone	Gournah	26th Dynasty	21.9	-	-	Touzeau et al. (2013)
30000157	Human	bone	Gournah	26th Dynasty	22.6	31.4	-13.6	Touzeau et al. (2013)
30000158	Human	bone	Gournah	26th Dynasty	21.5	30.4	-13.4	Touzeau et al. (2013)
30000160	Human	bone	Gournah	26th Dynasty	22.6	31.5	-13.6	Touzeau et al. (2013)
30000241	Human	bone	Gournah	26th Dynasty	22.4	32.4	-14.6	Touzeau et al. (2013)
30000248	Human	bone	Gournah	26th Dynasty	21.2	30.9	-14.0	Touzeau et al. (2013)
-	Ratite	bone		Present-day	15.8	23.6	-	Stanton-Thomas and Carlson (2004)
-	Ratite	bone		Present-day	14.7	22.7	-	Stanton-Thomas and Carlson (2004)
-	Ratite	bone		Present-day	14.1	24.4	-	Stanton-Thomas and Carlson (2004)
-	Ratite	bone		Present-day	14.9	23.6	-	Stanton-Thomas and Carlson (2004)
-	Ratite	bone		Present-day	14.1	22.6	-	Stanton-Thomas and Carlson (2004)
-	Ratite	bone		Present-day	13.8	23.0	-	Stanton-Thomas and Carlson (2004)
-	Ratite	bone		Present-day	13.5	22.1	-	Stanton-Thomas and Carlson (2004)
-	Ratite	bone		Present-day	13.6	22.1	-	Stanton-Thomas and Carlson (2004)
-	Ratite	bone		Present-day	13.6	23.0	-	Stanton-Thomas and Carlson (2004)
-	Ratite	bone		Present-day	13.6	24.3	-	Stanton-Thomas and Carlson (2004)
-	Ratite	bone		Present-day	13.9	23.1	-	Stanton-Thomas and Carlson (2004)
-	Ratite	bone		Present-day	13.8	24.3	-	Stanton-Thomas and Carlson (2004)

Table S5: published Oxygen and carbon isotope compositions of apatite carbonate ($\delta^{18}O_c$ and $\delta^{13}C_c$) and phosphate ($\delta^{18}O_p$) from mummified fish, cats and humans, as well as extant birds.

Reference:

Stanton-Thomas, K. J. & Carlson, S. J. Microscale $\delta^{18}O$ and $\delta^{13}C$ isotopic analysis of an ontogenetic series of the hadrosaurid dinosaur Edmontosaurus: implications for physiology and ecology. Palaeogeography, Palaeoclimatology, Palaeoecology 206, 257–287 (2004).

Touzeau, A. et al. Egyptian mummies record increasing aridity in the Nile valley from 5500 to 1500 yr before present. Earth and Planetary Science Letters 375, 92–100 (2013).
